# Diagnostic value of third-generation nanopore sequencing in extrapulmonary tuberculosis

**DOI:** 10.3389/fcimb.2024.1432446

**Published:** 2025-01-07

**Authors:** Chang Song, Chunyan Zhao, Yanrong Lin, Yingxing Nong, Aichun Huang, Shaoyong Xi, Xiaoying Wei, Chunmei Zeng, Shixiong Yang, Qingdong Zhu

**Affiliations:** ^1^ Department of Tuberculosis, The Fourth People’s Hospital of Nanning, Nanning, China; ^2^ Guangxi Medical University, Nanning, China; ^3^ Department of Medical, The Fourth People’s Hospital of Nanning, Nanning, China; ^4^ Department of Clinical Laboratory, The Fourth People’s Hospital of Nanning, Nanning, China; ^5^ Administrative Office, The Fourth People’s Hospital of Nanning, Nanning, China

**Keywords:** nanopore sequencing, extrapulmonary tuberculosis, diagnostic value, conventional methods, clinical microbiology

## Abstract

**Background:**

This study aimed to explore the accuracy of third-generation nanopore sequencing to diagnose extrapulmonary tuberculosis (EPTB).

**Methods:**

Samples were collected from the lesions of 67 patients with suspected EPTB admitted between April 2022 and August 2023. Nanopore sequencing, acid-fast bacilli (AFB) staining, DNA testing, and X-pert and mycobacterial cultures were performed. The sensitivity, specificity, positive predictive value (PPV), negative predictive value (NPV) and area under the receiver operating characteristic curve (AUC) were calculated for different diagnostic methods, and their diagnostic accuracies were compared.

**Results:**

Nanopore sequencing demonstrated the highest correct diagnosis rate among 50 positive EPTB cases, independently diagnosing 19 positive cases missed by conventional methods. Its sensitivity (62.00%), specificity (94.10%), PPV (96.90%), NPV (45.70%) and AUC (0.781, 95% CI: 0.67–0.89) were superior to those of conventional methods, such as AFB staining, DNA testing, X-pert, and solid culture, indicating its significantly efficient advantage in EPTB detection.

**Conclusion:**

Nanopore sequencing technology significantly outperforms conventional methods such as AFB staining, DNA testing, X-pert, and mycobacterial culture to diagnose EPTB, promising to improve the diagnosis of EPTB.

## 1 Introduction

Tuberculosis (TB), caused by *Mycobacterium tuberculosis*, is a chronic infectious disease that mainly affects the lungs and causes pulmonary tuberculosis (PTB) and has always been a major concern for the World Health Organization (Dong et al., 2022). Traditionally, TB has been associated with PTB. However, extrapulmonary tuberculosis has gradually increased, attracting widespread attention (Peto et al., 2009; Neelakantan et al., 2013). Studies have shown that EPTB accounts for 15% of tuberculosis infections, and it is more difficult to diagnose EPTB than traditional PTB based on radiological characteristics and clinical symptoms. Most cases require a biopsy to confirm diagnosis (Rodriguez-Takeuchi et al., 2019).

The complexity and challenges of EPTB lie in the fact that the lesions occur in tissues and organs other than the lungs and involve a wide range of manifestations. Common types of EPTB include lymph nodes, pleura, skin, ear, nose and throat, genitourinary system, pericardium, gastrointestinal tract, bones and joints, and the central nervous system (Muneer et al., 2019; Schaller et al., 2019; Al-Zanbagi and Shariff, 2021; Brehm and Terhalle, 2023). EPTB is prone to misdiagnosis, primarily because it lacks typical clinical symptoms compared to PTB. Patients may present with symptoms, such as fatigue, fever, weight loss, and local masses. These symptoms may be mistakenly attributed to other diseases, such as cancer, by doctors due to the lack of specific symptoms, delaying the diagnosis of EPTB (Aisenberg et al., 2005). Additionally, EPTB-affected tissues or organs, such as the lymph nodes, meninges, abdomen, bones, and joints, are often difficult to sample. Compared to PTB, there are limited methods for obtaining non-invasive samples, thus delaying patient diagnosis. In some cases, invasive surgery or other procedures may be required to obtain tissue samples, increasing patient risk and discomfort (Norbis et al., 2014). Furthermore, unlike PTB, the sites of EPTB infection often have a lower bacterial load, presenting that the positive rate of conventional bacterial culture or microscopy methods decreases (Jain, 2011). Additionally, current standard TB diagnostic tools are mainly designed for PTB and may not have the same sensitivity and specificity for EPTB. Therefore, doctors must rely on other diagnostic techniques, such as radiological examination, pathological evaluation, microbiological testing, and molecular biology techniques, to improve the accuracy of diagnosis, and sometimes rely on histopathological evidence for final diagnosis. However, these diagnostic methods inevitably have defects, such as long processing time and low accuracy, greatly affecting disease monitoring and timely diagnosis and treatment. Based on the current diagnostic status of EPTB, there is an urgent need for a new, efficient, and accurate molecular biology method to improve the diagnosis of EPTB.

Third-generation nanopore sequencing technology is an advanced genomic sequencing technology that measures changes in the current as DNA or RNA molecules pass through nanopores to achieve sequence reading (Sun et al., 2020). This technology has significant advantages in rapidly diagnosing infectious diseases due to its high throughput, real-time capabilities, and long read lengths (Żmieńko and Satyr, 2020). The read lengths of third-generation nanopore sequencing may be tens of thousands of base pairs, significantly limiting second-generation sequencing (Meslier et al., 2022). It avoids the problems of primer bias and polymerase chain reaction (PCR) reproducibility caused by PCR primers because it does not require PCR, making the sequencing results more reliable (Chapman et al., 2023). Additionally, the high coverage of third-generation sequencing technology can cover the entire genome, making it possible to comprehensively analyze the genome of *M. tuberculosis*. Therefore, third-generation nanopore sequencing technology has shown great potential for diagnosing TB, which is highly infectious and time-consuming, using conventional methods.

Currently, third-generation nanopore sequencing technology has achieved a series of results for diagnosing PTB. However, there remains a lack of research on applying third-generation nanopore sequencing technology to EPTB. This study aimed to conduct a prospective cohort analysis and explore its value in diagnosing EPTB.

## 2 Material and methods

### 2.1 Subject inclusion and study design

This study included patients with suspected EPTB who were admitted and treated at the Fourth People’s Hospital of Nanning, Guangxi Zhuang Autonomous Region, between April 2022 and August 2023. Additionally, electronic medical records were used to collect information on patients’ gender, age, underlying diseases, and medical history. Moreover, the results of acid-fast staining, DNA testing, X-pert testing, and mycobacterial cultures were collected from the enrolled patients. To ensure compliance with regulations and ethical requirements, the study was approved by the Human Research Ethics Committee of the Fourth People’s Hospital of Nanning, Guangxi Zhuang Autonomous Region (Approval No: [2023]24), and informed consent was obtained from all participants or their legal guardians. To ensure the integrity and scientific validity of the study, clinical specimens that did not undergo acid-fast staining, mycobacterial culture, X-pert MTB/RIF, and nanopore sequencing were excluded. The final diagnostic criteria for EPTB involved comprehensive clinical assessments, including a detailed medical history and physical examination, laboratory tests such as culture and PCR, radiological and histopathological examinations, and molecular biology test results. The key points included positive samples from the lesion sites, liquid samples, histopathological changes, and results based on molecular detection and clinical confirmation. Positive response to anti-TB treatment is an important diagnostic indicator.

### 2.2 Sample collection

In this study, samples were collected following the requirements of “WS/T 640-2018 Collection and Transportation of Clinical Microbiological Test Specimens” (China NHCotPsRo, 2018) formulated by the National Health Commission of the People’s Republic of China and the “Chinese X-pert Consensus on Standardized Collection and Submission of Clinical Microbiological Specimens” (Association, 2017) formulated by the Hospital Infection Control Branch of the Chinese Preventive Medicine Association. Strict aseptic techniques were employed during the sampling, and physical sterilization methods, such as autoclaving, were used instead of chemical disinfectants. Various samples, including cerebrospinal fluid, pleural fluid, abscess fluid, pericardial effusion, peripheral blood, bone marrow, tissue samples, and urine, were collected before anti-infective treatment and were quickly tested. After collection, the samples were divided into multiple parts for different testing methods. For each testing method including acid - fast staining, mycobacterial solid culture, X-pert MTB/RIF, and nanopore sequencing, identical aliquots of the samples were used whenever possible.

### 2.3 Acid-fast staining

In this study, fluorescent auramine-O staining was used for the acid-fast staining. A direct smear was prepared using a new glass slide with a frosted end and no scratches, which was degreased with 95% ethanol, dried, and cleaned before use. The experimental and specimen numbers were marked on the frosted end of the slide to ensure consistency with the numbers in the sputum box. The sputum specimen container was opened in a biosafety cabinet to prevent aerosol formation or spilling. Approximately 0.05 mL of caseous, purulent, or suspicious parts of the sputum specimen were picked and spread evenly on the slide’s frosted side to form an oval sputum film measuring 10 mm × 20 mm. The slide was placed upside down in a biosafety cabinet and allowed to air-dry for approximately 30 min before staining and microscopic examination. The staining solution (Zhuhai Beso Biotechnology Co., Ltd.) consisted of three bottles (4 × 250 mL) containing auramine-O, acidic alcohol, and 5% potassium permanganate solutions. After staining, the acid-fast bacilli appeared as slightly curved rods.

### 2.4 DNA testing

In this study, an advanced DNA testing kit combining dual PCR with TaqMan probe technology was used for DNA analysis. This kit specifically includes primer and probe sequences targeting *M. tuberculosis* complex (MTBC) and non-tuberculous mycobacteria (NTM), ensuring accurate targeting and identification. The presence of MTBC and NTM was detected by monitoring signal changes in different fluorescence channels. The key equipment used in the experiment included Applied Biosystems 7500, Shanghai Hongshi SLAN-96S, and SLAN-96P real-time fluorescence quantitative PCR instruments. The testing kit was provided by Chengdu Boao Jingchi Biotechnology Co., Ltd. (based on the PCR-fluorescent probe method).

### 2.5 X-pert MTB/RIF testing

This testing method involves processing patient samples with specialized reagents and adding the sample to the X-pert cartridge (Steingart et al., 2014). X-pert MTB/RIF combines semi-nested real-time fluorescence multiplex PCR and microfluidic technology to achieve fully automated detection of M.tuberculosis.

### 2.6 Mycobacterial culture

In this study, a modified Roche medium (Zhuhai Beso Technology Co., Ltd., 50 vials/box) was used for *mycobacterial* culture. The sample processing steps included adding 4% NaOH solution, vortexing, incubating at room temperature, adding pH 7.2 phosphate buffer, and centrifuging. Positive results were reported immediately after confirmation using a smear examination. Observations were conducted three and seven days after inoculation and weekly after that. Positive results were reported immediately after confirmation using a smear examination. If the result was positive within seven days, it was considered rapid growth; otherwise, it was considered slow growth. Negative results were reported after eight weeks.

### 2.7 Nanopore sequencing

In this study, nanopore sequencing was conducted by Hangzhou Shengting Medical Technology Co. Ltd. Before sequencing analysis, preparatory work was performed, including processing reagents, such as proteinase K, lysozyme, and plasmids, and adding the samples to the processing. The samples were added to the reagents for processing during nucleic acid extraction. After incubation, the precipitate was centrifuged with anhydrous ethanol, and the pellet was placed in an Eppendorf (EP) tube. Subsequently, magnetic beads were added for oscillation and incubation. Before discarding the supernatant, 1XWB solution and ethanol were added. After processing the EP tubes, magnetic beads were activated and incubated. The nucleic acid quality was measured using a Qubit 4.0 DNA concentration measuring instrument to ensure qualification. Subsequently, the PCR system was prepared, and a nucleic acid sample was added. After verification, the reaction tube was placed in the PCR instrument for operation. After PCR, purification was performed, and the DNA library was extracted. Barcode labeling and another round of PCR amplification were performed on the multiplex PCR products and stored. Subsequently, the PCR products were purified and quality-checked, and products with different labels were mixed and operated according to the nanopore sequencing library preparation kit. After library preparation, GridION sequencing was performed. After data collection, the data were subjected to quality filtering and alignment, and pathogenic microorganism species and drug-resistance gene analyses were generated.

### 2.8 Data processing and analysis

In this study, the SPSS 25.0 software package was used for statistical processing and data analysis. A 2×2 contingency table was constructed to comprehensively evaluate the sensitivity, specificity, positive predictive value, negative predictive value and agreement ratio. The McNemar chi-square test was used as the statistical method to compare the differences in diagnostic efficacy between nanopore sequencing, acid-fast staining, DNA testing, X-pert testing, and mycobacterial culture. When the P-value was less than 0.05, the difference reached statistical significance.

## 3 Results

### 3.1 Characteristics of the subjects

**Table 1** presents the basic information of the 67 study subjects and the clinical sample types. Among the 50 cases diagnosed with TB, 33 were males (66%), and 17 were females (34%), with an age distribution of 48.52 ± 2.85 years. Of the 17 cases without confirmed TB, 14 were males (82.35%), and three were females (17.65%), with an age distribution of 59.12 ± 4.20 years. Among all subjects, three cases were comorbid with diabetes, and eight cases were comorbid with HIV.

**Table 1:** Basic information of the subjects and test samples.

Characteristics	Number
Total	67
TB	50(Male:33,Famale:17)
Non-TB	17(Male:14,Famale:3)
Age(years)(mean± SD)	
TB	48.52 ± 2.85
Non-TB	59.12 ± 4.20
Gender	
Male	47
Famale	20
Other Diseasese	
Diabetes	3
HIV	8
Classification of specimens	
Penetrating the tissues	5
Cerebrospinal fluid	20
Aspirating pus from an abscess.	12
Pericardial effusion.	2
Fresh assembly	5
Pleural effusion	16
Marrow	1
Urine	1
Peripheral blood	2
Pleural tissue & pleural effusion mixture sample	1
Marrow & blood mixture sample	1
tissues Penetrated & aspirating pus from an abscess mixture sample	1

Characteristics include Age, Gender, and Classification of specimens.

**Table 2** shows the final confirmed sites of EPTB. Joint tuberculosis was recorded in only two cases among females, with no reported cases in males. Tuberculous meningitis/encephalitis was present in 14 males and only two females, 16 cases in total. Tuberculous pleurisy was reported in 16 patients, 12 males and four females. Chest wall tuberculosis was found only in males, with two cases in females. Lymph node tuberculosis was reported in seven patients, including three males and four females. Tuberculous pericarditis was present in only two females. Psoas abscess and thoracolumbar abscess due to tuberculosis were reported in one female, without records in males. Paravertebral tuberculosis was reported in one male and one female. One male patient reported a gluteal abscess, without reports in females.

**Table 2:** Diagnostic site.

Affected area	Male	Famale	Total
Tuberculosis of the joints	0	2	2
Tuberculous encephalitis/meningitis	14	2	16
Tuberculous pleurisy	12	4	16
Tuberculosis of the chest wall	2	0	2
Tuberculous pericarditis	0	2	2
Tuberculosis of the lymph nodes	3	4	7
Tuberculous abscess of psoas major muscle	1	0	1
Paravertebral tuberculosis	1	1	2
Tuberculosis of the thoracic mediastinum	0	1	1
Tuberculosis of left lumbar vertebra	0	1	1

### 3.2 Detection results of nanopore sequencing, acid-fast staining, DNA testing, X-pert, and mycobacterial solid culture

**Figure 1:**
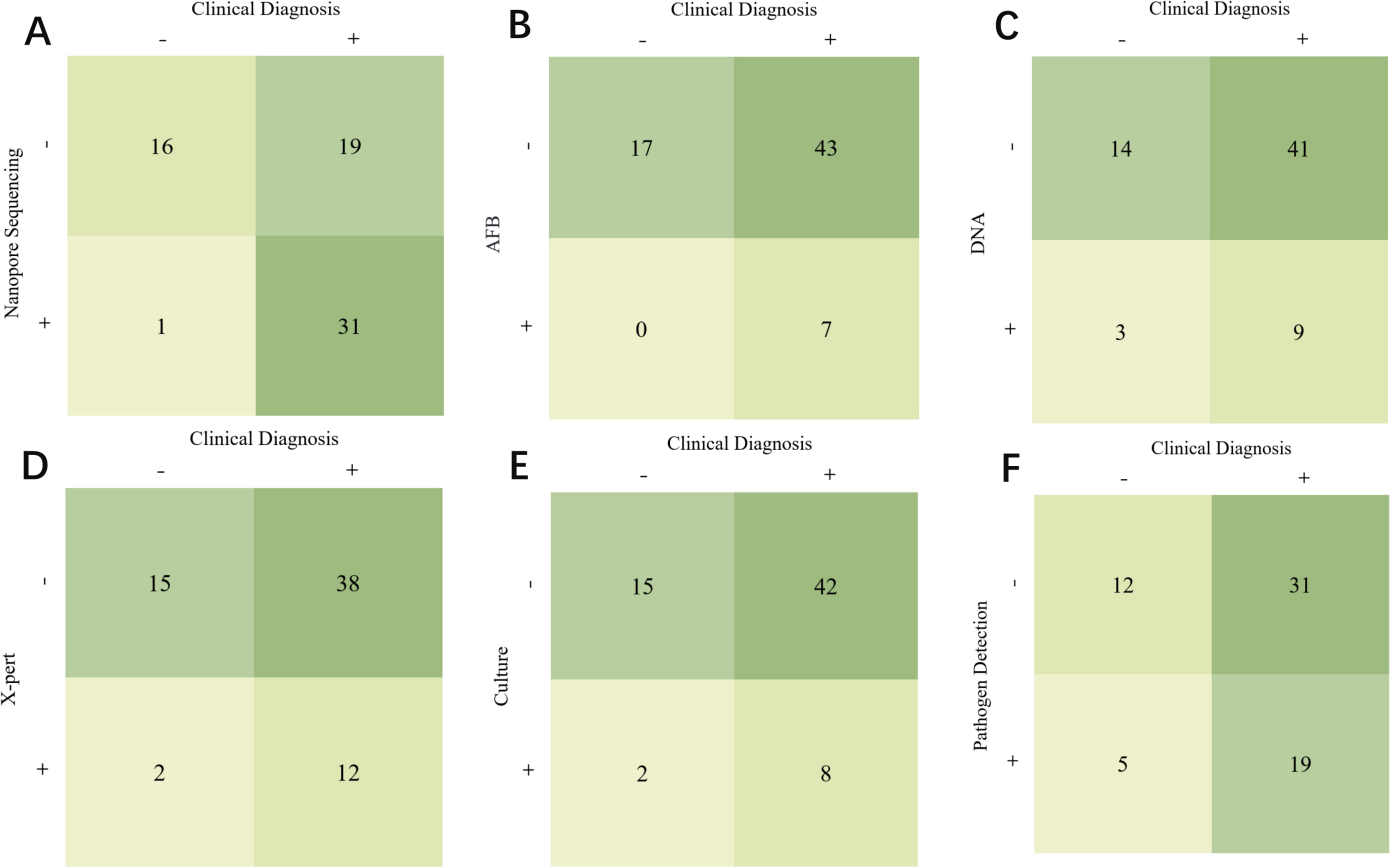
Comparison of different detection methods and clinical diagnosis. **(A)** Comparison of Clinical Diagnosis and Nanopore Sequencing. **(B)** Comparison of Clinical Diagnosis and AFB. **(C)** Comparison of Clinical Diagnosis and DNA. **(D)** Comparison of Clinical Diagnosis and X-pert. **(E)** Comparison of Clinical Diagnosis and Culture. **(F)** Comparison of Clinical Diagnosis and Pathogen Detection.

**Figure 1** compares different detection methods with clinical diagnosis and indicates that nanopore sequencing has the highest correct diagnostic rate among the 31 TB cases. In contrast, acid-fast staining, DNA testing, X-pert, and mycobacterial solid culture, the four conventional detection methods, missed up to 43, 41, 38, and 42 positive cases, respectively. Notably, these conventional methods collectively missed 31 positive cases.

**Figure 2:**
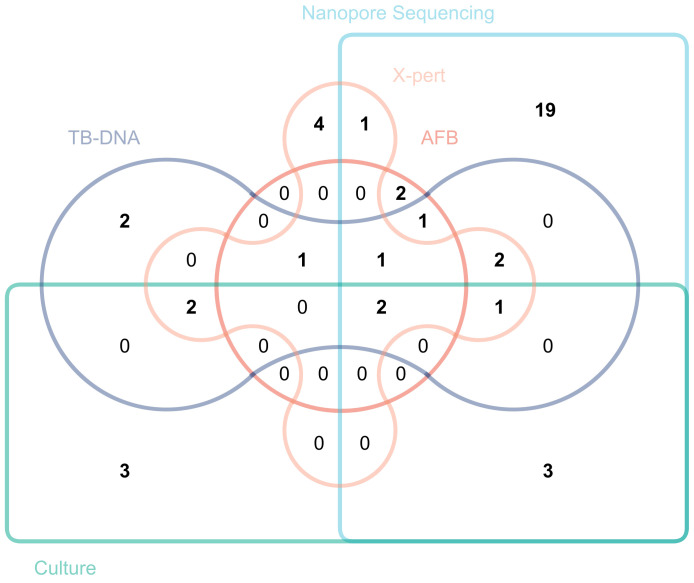
Venn diagram with a positive diagnosis of extrapulmonary tuberculosis.

**Figure 2** plots a Venn diagram to demonstrate the overlap and distribution of positive results among several detection methods. The results showed that third-generation nanopore sequencing technology independently diagnosed 19 positive cases not diagnosed by other detection methods.

### 3.3 Diagnostic performance of nanopore sequencing, acid-fast staining, DNA testing, X-pert, and mycobacterial solid culture

**Table 3** shows the statistical analysis of the specificity, sensitivity, NPV, PPV and area under the receiver operating characteristic curve (AUC) values of various diagnostic methods. The indicators for nanopore sequencing were 94.10%, 62.00%, 45.70%, 96.90%, 0.416, and 0.781 (95% CI: 0.67–0.89), respectively. For AFB, the values were 100.00%, 14.00%, 28.30%, 100.00%, 0.076, and 0.570 (95% CI: 0.42–0.72), respectively. For DNA testing, these values were 82.40%, 18.00%, 25.50%, 75.00%, 0.002, and 0.502 (95% CI: 0.34–0.66), respectively. For X-pert, the values were 88.20%, 24.00%, 28.30%, 85.70%, 0.072, and 0.561 (95% CI: 0.41–0.71), respectively. For solid culture, the values were 88.20%, 16.00%, 26.30%, 80.00%, 0.024, and 0.521 (95% CI: 0.36–0.68), respectively.

**Table 3:** Diagnostic efficacy of nanopore sequencing accay, AFB, DNA testing, X-pert and TB culture.

	Specificity	Sensitivity	NPV	PPV	AUC
Nanopore Sequencing	94.10%	62.00%	45.70%	96.90%	0.781(95%CI:0.67-0.89)
AFB	100.00%	14.00%	28.30%	100.00%	0.570(95%CI:0.42-0.72)
DNA	82.40%	18.00%	25.50%	75.00%	0.502(95%CI:0.34-0.66)
X-pert	88.20%	24.00%	28.30%	85.70%	0.561(95%CI:0.41-0.71)
Culture	88.20%	16.00%	26.30%	80.00%	0.521(95%CI:0.36-0.68)

### 3.4 Diagnostic performance of nanopore sequencing combined with acid-fast bacillus smear, DNA detection, X-pert, and mycobacterial culture

From the perspective of ROC, the diagnostic method combining AFB testing for tuberculosis with nanopore sequencing has the highest predictive value for clinical diagnosis, with an AUC value of 0.791 (95% CI: 0.68-0.90). Next is X-pert & nanopore sequencing (0.772 (95% CI: 0.64-0.90)), mycobacterial solid culture & nanopore sequencing (0.742 (95% CI: 0.61-0.88)), and DNA & nanopore sequencing (0.712 (95% CI: 0.57-0.85)) (**Table 4**).

**Table 4:** Diagnostic performance of nanopore sequencing combined with acid-fast bacillus smear, DNA detection, X-pert, and mycobacterial culture.

	Specificity	Sensitivity	NPV	PPV	AUC
Nanopore Sequencing & AFB	94.10%	64.00%	47.10%	97.00%	0.791(95%CI:0.68-0.90)
Nanopore Sequencing & DNA	76.50%	66.00%	43.30%	89.20%	0.712(95%CI:0.57-0.85)
Nanopore Sequencing & X-pert	82.40%	72.00%	50.00%	92.30%	0.772(95%CI:0.64-0.90)
Nanopore Sequencing & Culture	82.40%	66.00%	45.20%	91.70%	0.742(95%CI:0.61-0.88)

## 4 Discussion

Tuberculosis is one of the deadliest diseases globally, particularly in low- and middle-income countries, despite the significant mortality caused by COVID-19 in recent years (Falzon et al., 2023). According to new data, the COVID-19 pandemic has markedly decreased reported TB cases and Bacillus Calmette-Guérin (BCG) immunization, exacerbating the spread of the disease and increasing susceptibility among populations (Shariq et al., 2022). However, TB primarily affects the lungs, and EPTB may occur in up to 15% of patients. Additionally, studies have shown that 15%–20% of immunocompetent patients and over 50% of HIV-infected individuals may develop EPTB via exogenous transmission (Shariq et al., 2022). The incidence of EPTB has recently shown a gradual increase worldwide. Besides the diagnostic challenges mentioned earlier, when considering the diagnosis of EPTB, the possibility of EPTB cannot be completely ruled out even if a patient’s chest X-ray results are normal or laboratory tests show negative results. Particularly when HIV positive is detected in patients, a high degree of suspicion for this diagnosis is required (Le Roux and Vlok, 2021). Several diagnostic methods have failed to meet the diagnostic needs of EPTB. Therefore, there is an urgent need for a more accurate, rapid, and effective diagnostic method to facilitate the early detection of EPTB.

Third-generation nanopore sequencing technology has recently garnered significant attention for its rapid and high-resolution sequencing capabilities. This technology enables single-molecule sequencing, thereby avoiding the influence of PCR and resulting in lower error rates and longer read lengths. Therefore, it has significant advantages in areas, such as complex genome recombination, structural variation, and mutation detection (Mariner-Llicer et al., 2021). Furthermore, the high efficiency of nanopore sequencing technology provides robust support for tracking pathogen variation and trends. Monitoring genetic variation typically requires intensive sample analysis and rapid data processing. Nanopore sequencing technology, with its rapid data output capability, is ideal for this task (Riaz et al., 2021). This is particularly important for developing personalized therapies and vaccines to help scientists and doctors combat constantly changing viruses and bacteria. These advantages have led to the widespread application of nanopore sequencing technology for rapidly diagnosing infectious diseases, especially TB, which is highly infectious and clinically time-consuming (Gómez-González et al., 2022). Previous studies have shown that nanopore sequencing technology has excellent diagnostic value for the early diagnosis of PTB. However, these studies have focused on using clinically standardized samples, such as bronchoalveolar lavage fluid or sputum, lacking evidence supporting the equal value of nanopore sequencing technology in diagnosing EPTB when faced with complex and diverse samples from extrapulmonary sites. Therefore, in this study, samples from 67 cases suspected of EPTB were collected, and nanopore sequencing, acid-fast staining, DNA testing, X-pert, and mycobacterial solid cultures were performed on lesions from different sites to assess their actual value in clinical diagnosis. We hope to provide clinicians with more accurate diagnostic tools for handling complex and diverse samples by comparing the diagnostic results of different samples, thereby improving diagnostic accuracy and treatment outcomes in clinical practice.

In this study, 50 patients were ultimately diagnosed with non-PTB. The study results showed that while there were no false-positive cases for diagnosing *M. tuberculosis*, only seven positive patients were successfully detected, leaving another 43 positive patients undiagnosed by AFB. Extrapulmonary samples with low bacterial load, insufficient sensitivity of detection methods, microscope observation errors, low bacterial load during early disease stages or treatment periods, and differences in host immune responses can all potentially lead to false-negative results in acid-fast bacilli detection. The specificity, sensitivity, NPV, PPV and AUC value of this diagnostic method were 100.00%, 14.00%, 28.30%, 100.00%, and 0.570 (95% confidence interval: 0.42-0.72), consistent with the findings of Sun et al (Sun et al., 2021). Although mycobacterial solid culture is considered the gold standard for diagnosis, it did not demonstrate an excellent diagnostic value in this study. Our research indicated that mycobacterial solid culture missed 42 positive cases, with specificity, sensitivity, NPV, PPV and AUC values of 88.20%, 16.00%, 26.30%, 80.00%, 0.024, and 0.521 (95% CI: 0.36-0.68). In contrast, Yang et al. reported higher diagnostic accuracy of mycobacterial solid culture in the diagnosis of PTB, with specificity, sensitivity, NPV, PPV, and AUC values of 100%, 18.6%, 29.1%, 100%, and 0.593, respectively, surpassing its performance in the diagnosis of EPTB in this study (Yang et al., 2023). Molecular detection methods such as TB-DNA and X-pert, known for their high accuracy, did not demonstrate the expected significant advantages for diagnosing non-PTB in this study. This could be closely related to factors such as the low bacterial load in extrapulmonary samples in this study.

Third-generation nanopore sequencing technology exhibited excellent diagnostic performance in analyzing 50 positive cases, successfully detecting 31 cases with only one false positive. Regarding diagnostic indicators, nanopore sequencing demonstrated a specificity of 94.10%, a sensitivity of 62.00%, an NPV of 45.70%, a PPV of 96.90% and an AUC value of 0.781. Compared to previous studies on the diagnosis of PTB using nanopore sequencing, Zhou et al. reported specificity, sensitivity, NPV, PPV, and AUC values of 84.85%, 90.70%, 82.35%, 92.13%, and 0.88, respectively (Zhou et al., 2024). Yang et al. found the corresponding values of 95.4%, 85.3%, 68.3%, 98.2%, and 0.903 (Yang et al., 2023). Yu et al. provided the following data: 97.9%, 94.8%, 88.7%, 99.1%, and 0.96 (Yu et al., 2022). These data suggest that extrapulmonary samples may have influenced the performance of the nanopore sequencing technology to some extent, highlighting a variable that needs to be addressed and controlled in future research. Nanopore sequencing technology has made significant strides in reducing missed diagnoses, compared to other detection methods (AFB had 43 cases, DNA detection had 41 cases, X-pert detection had 38 cases, and culture method had 42 cases; while nanopore sequencing had only 19 cases), there has been a substantial decrease, yet this issue should not be overlooked. Molecular biology detection methods typically only effectively detect MTB when its content in the sample exceeds the detection limit. Therefore, when the MTB content in the sample is below the detection limit, it may lead to some missed detections of MTB. Secondly, improper operations in sample collection, transportation, and pretreatment processes, including insufficient monitoring during sequencing, may all result in missed diagnoses. This indicates that in the future, researchers should focus on improving this area, which includes optimizing the sample collection and processing procedures to ensure the integrity and stability of the samples throughout the process; enhancing laboratory personnel training to improve their proficiency in sample handling and detection technologies; and improving detection methods to make them more sensitive to samples with low concentrations of pathogens. Nevertheless, regarding reducing misdiagnosis rates and increasing the detection rate of hidden cases, third-generation nanopore sequencing technology has undoubtedly demonstrated significant superiority over traditional detection methods, thereby marking a major advancement in TB diagnosis. This study emphasized the practical value of nanopore sequencing technology, and another crucial advantage lies in its processing efficiency. In diagnosing EPTB, nanopore sequencing technology can rapidly complete sample collection for final report generation within a day. This time efficiency is significant, especially for patients requiring urgent intervention, as rapid diagnosis translates into timely treatment opportunities, greatly improving the likelihood of patient recovery and reducing the impact of the disease on their quality of life. Therefore, this technology has revolutionized diagnostic capabilities and significantly enhanced the efficiency of disease management and patient care. In summary, nanopore sequencing technology may be a powerful alternative to the aforementioned methods for diagnosing EPTB.

This study had certain limitations. First, the study was based on a relatively small sample size, limiting the generalizability and universality of the results. Future research needs to expand the sample size and consider factors, such as multicenter and multiethnicity, to validate the reliability and stability of nanopore sequencing technology in diagnosing EPTB. Second, as some patients had received anti-TB treatment at other hospitals before participating in this study, their conditions may have improved to some extent, or drug resistance may have developed. In such cases, the diagnostic accuracy of nanopore sequencing technology for their conditions interferes with and affects the objectivity of the study results. Additionally, differences in sample types, sample quality, and operational technical levels may influence the performance of the nanopore sequencing technology. Therefore, further optimization and improvement in the application of this technology in different contexts are needed to ensure its stable and reliable application in clinical diagnosis.

Overall, we are optimistic about the positive performance of nanopore sequencing technology for diagnosing EPTB. We must also acknowledge the study’s limitations and technological challenges. Nanopore sequencing technology, with its processing speed and ongoing optimization of technology and algorithms, may provide a solid and broad foundation for the precise diagnosis of EPTB and personalized treatment in the near future.

## 5 Conclusion

Nanopore sequencing demonstrated excellent diagnostic value in suspected TB diagnoses from extrapulmonary samples, significantly outperforming acid-fast staining, DNA testing, X-pert, and M. tuberculosis solid cultures. Nanopore sequencing technology shows enormous potential in EPTB diagnosis, offering a novel possibility for diagnosis and pathogen monitoring with its rapid and efficient characteristics.

## Data Availability

The original contributions presented in the study are publicly available. This data can be found here: https://ngdc.cncb.ac.cn, accession number PRJCA033908.
